# Supporting data for the characterization of PNA–DNA four-way junctions

**DOI:** 10.1016/j.dib.2015.10.013

**Published:** 2015-10-22

**Authors:** Douglas Iverson, Crystal Serrano, Ann Marie Brahan, Arik Shams, Filbert Totsingan, Anthony J. Bell

**Affiliations:** a Department of Chemistry and Biochemistry, The University of Southern Mississippi, Hattiesburg, MS, USA; b Department of Chemistry, New York University, New York, NY, USA

## Abstract

Holliday or DNA four-way junctions (4WJs) are cruciform/bent structures composed of four DNA duplexes. 4WJs are key intermediates in homologous genetic recombination and double-strand break repair. To investigate 4WJs *in vitro*, junctions are assembled using four asymmetric DNA strands. The presence of four asymmetric strands about the junction branch point eliminates branch migration, and effectively immobilizes the resulting 4WJ. The purpose of these experiments is to show that immobile 4WJs composed of DNA and peptide nucleic acids (PNAs) can be distinguished from contaminating labile nucleic acid structures. These data compare the electrophoretic mobility of hybrid PNA–DNA junctions *vs*. i) a classic immobile DNA 4WJ, J1 and ii) contaminating nucleic acid structures.

**Specifications Table**

**Table:** 

Subject area	*Biochemistry and biophysics*
More specific subject area	*Nucleic acid biochemistry*
Type of data	*Nondenaturing polyacrylamide gel electrophoresis*
How data was acquired	*Scanned with Typhoon 9400 Phosphorimager*
Data format	*Scanned gel images*
Experimental factors	*4WJs and DNAs are assembled by mixing, lyophilizing and suspending in annealing buffer [50* *mM TrisHCl (pH 7.5), 1* *mM MgCl*_*2*_*].*
*Immobile 4WJs travel with slower mobility than mobile 4WJs and contaminating nucleic acid structures.*
Experimental features	*One DNA strand in each sample contains a flourescein label that is quantified via Phoshorimager analysis.*
Data source location	*The University of Southern Mississippi, Hattiesburg, MS*
Data accessibility	*The data are supplied with this article.*

**Value of the data**

• The technique provides an approach to confirm the formation of previously uncharacterized 4WJs.
• The technique provides an approach to characterize the global structural features of immobile 4WJs composed of DNA and PNA.
• The technique provides an exhaustive approach to investigate different combinations of potentially contaminating DNA structures *vs*. immobile hybrid PNA–DNA 4WJs.

## 1 Data

**Fig. 1:**
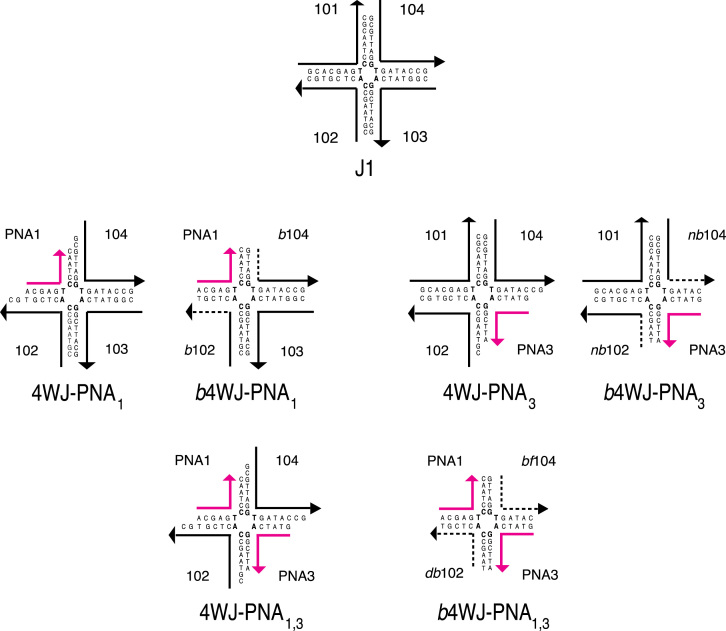
DNA junction, J1, and hybrid PNA–DNA four-way junctions.

**Fig. 2:**
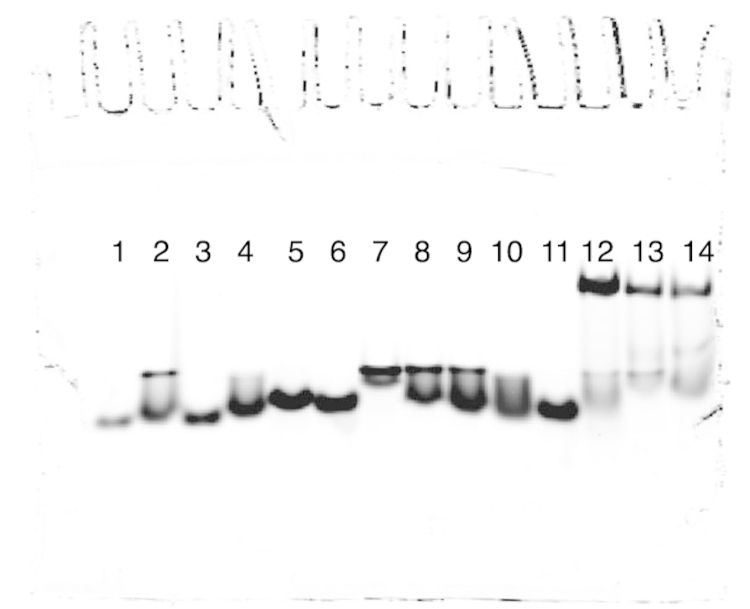
Comparison of multi-DNAs *vs.* J1, 4WJ:PNA_1_ and *b*4WJ:PNA_1_.

**Fig. 3:**
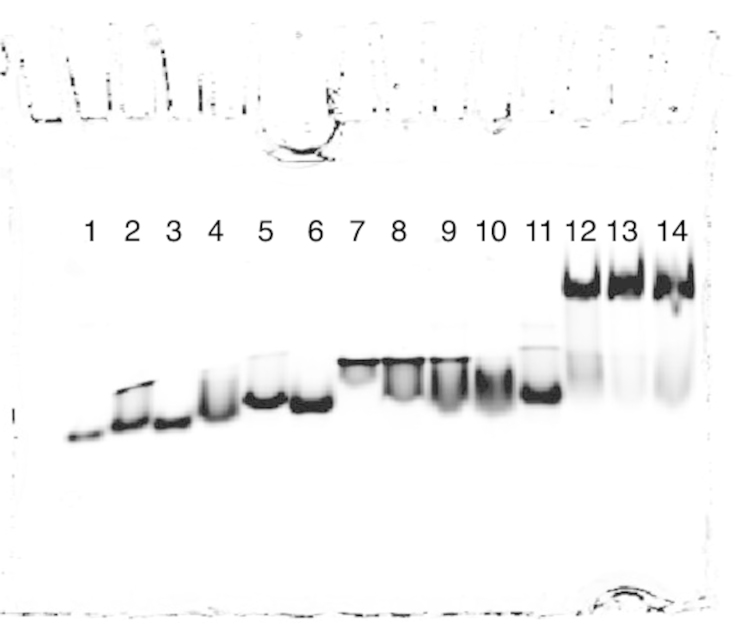
Comparison of multi-DNAs *vs.* J1, 4WJ:PNA_3_ and *b*4WJ:PNA_3_.

**Fig. 4:**
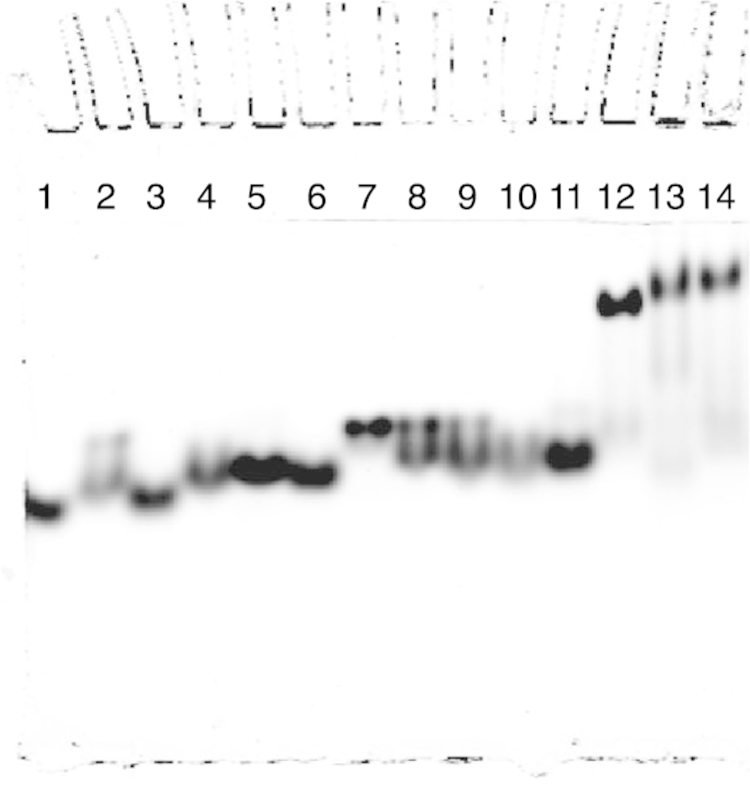
Comparison of multi-DNAs *vs.* J1, 4WJ:PNA_1,3_ and *b*4WJ:PNA_1,3_.

The nucleic acid sequence of the DNA control, J1 and each hybrid PNA–DNA junction is displayed in Fig. 1 [1]. The data in Fig. 2, Fig. 3, Fig. 4 display the electrophoretic mobility patterns of six hybrid PNA–DNA 4WJs *vs.* i) J1 and ii) different combinations of potentially contaminating strands of DNA. The contaminating strands are composed of single strands used to form J1. The data are based on the protocol used by Kallenbach and Seaman to characterize, J1 [2], [3]. In our previous study, each hybrid PNA–DNA 4WJ is evaluated *vs.* J1 [4].

## 2 Experimental design, materials and methods

### 2.1 Preparation of 4WJs and multi-DNAs

Each 4WJ is formed by lyophilizing a mixture of one fluorescein labeled strand (25 μM) with 5-fold excess of three unlabeled strands (125 μM). The multi-DNAs samples are formed by lyophilizing a mixture of a fluorescein labeled strand with 5-fold excess unlabeled stands. The flourescein strands are denoted with an asterisk (below). Each pellet is suspended in 50 mM Tris-HCl (pH 7.5) and 1.0 mM MgCl_2_, incubated at 95 °C for 2 min and cooled to room temperature for 12–16 h. To determine the purity of each 4WJ, 2.5 μM of each sample is loaded onto 15% mini-PROTEAN nondenaturing polyacrylamide gels (BioRad) and run for 1–5 h (4 °C). The gel running buffer is composed of 0.5× TBE·MgCl_2_ buffer (45 mM Trisma, 45 mM boric acid, 1.0 mM EDTA and 1 mM MgCl_2_), pH 7.6. The gels are subsequently scanned with a Typhoon 9400 Phosphorimager. A list of the corresponding oligonucleotides of each multi-DNA sample and 4WJs are shown below.

Fig. 2: Lanes 1–14: 101^*^, 101^*^–102, 101^*^–103, 101^*^–104, 102–103^*^, 102–104^*^, 103–104^*^, 101^*^–102–103, 101^*^–102–104, 101^*^–103–104, 102–103-–104^*^, J1(101^*^), 4WJ:PNA_1_(103^*^) and *b*4WJ:PNA_1_(103^*^).

Fig. 3: Lanes 1–14: lanes 1–14: 101^*^, 101^*^–102, 101^*^–103, 101^*^–104, 102–103^*^, 102–104^*^, 103–104^*^, 101^*^–102–103, 101^*^–102–104, 101^*^–103–104, 102–103–104^*^, J1(101^*^), 4WJ:PNA_3_(101^*^) and *b*4WJ:PNA_3_(101^*^).

Fig. 4: Lanes 1–15: lanes 1–14: 101^*^, 101^*^–102, 101^*^–103, 101^*^–104, 102–103^*^, 102–104^*^, 103–104^*^, 101^*^–102–103, 101^*^–102–104, 101^*^–103–104, 102–103–104^*^, J1 (101^*^), 4WJ:PNA_1,3_(104^*^), *b*4WJ:PNA_1,3_(bf104^*^) and *b*4WJ:PNA_1,3_(bf104^*^).

## 3 Data interpretation

The data provide a direct method to compare the mobility of immobile hybrid PNA–DNA junctions *vs.* a DNA control junction (J1) and potentially contaminating multi-PNAs. In each gel (Fig. 2, Fig. 3, Fig. 4); the single strand control (101) is loaded in lane 1, the contaminating strands are loaded in lanes 2–11, the DNA control, J1 is loaded in lane 12, and the hybrid PNA–DNA junctions are loaded in lanes 13 and 14. In each case, the hybrid 4WJ with a DNA overhang(s) is loaded in lane 13 and the blunt-ended construct is loaded in lane 14.
